# The correlation between ultrasonographic morphology and structure of the left atrial appendage, blood flow velocity, and plasma galectin-3 levels with thrombus formation in the left atrial appendage of patients with atrial fibrillation

**DOI:** 10.5937/jomb0-48509

**Published:** 2024-06-15

**Authors:** Linghui Zhao, Min Li, Yuchen Zhang, Wenrui Tang, Dawei Huang, Guanjin Zhou, Bo Zhu, Zhiqi Han, Dingyue Zhu

**Affiliations:** 1 Huai 'an Fifth People's Hospital, Department of Cardiovascular Medicine, Huaian, China; 2 Lianshui County Traditional Chinese Medicine Hospital, Huaian, China; 3 Capital Medical University, Beijing Anzhen Hospital, Department of Cardiology, Beijing, China; 4 Lianshui County Hospital of Traditional Chinese Medicine, Department of Cardiology, Huai'an City, Jiangsu Province, China; 5 Guilin Medical University, Guilin, China; 6 Nanjing Medical University, Nanjing, China

**Keywords:** left atrial appendage morphology, blood flow velocity, galectin-3, atrial fibrillation, thrombus in the left atrial appendage, morfologija apendiksa leve atrijale, brzina krvotoka, galektin-3, atrijalna fibrilacija, tromb u dodatku leve pretkomore

## Abstract

**Background:**

To explore the correlation between left atrial appendage morphology, blood flow velocity and plasma galectin-3 and thrombosis in patients with atrial fibrillation.

**Methods:**

Patients with atrial fibrillation who received treatment and completed ultrasound examination in hospital from 2022 to December 2023 were enrolled. According to whether there was left atrial appendage thrombosis, the patients were divided into a control group (no left atrial appendage thrombosis was found) and a study group (left atrial appendage thrombosis was found). The morphology and structure of the left atrial appendage, blood flow velocity and plasma galectin-3 level were recorded exploring its correlation with left atrium thrombosis.

**Results:**

A total of 330 patients with atrial fibrillation were enrolled, including 278 in the control group and 52 in the study group. Left group and the control group of morphological structure differences (P < 0.05). The main lobe length, ostial area, longest diameter, shortest diameter, left atrial appendage volume and left atrial volume in the study group were higher than those in the control group (P < 0.05). The left atrial appendage emptying velocity, filling velocity and left ventricular ejection fraction of the study group were lower than those of the control group, and the left ventricular end-diastolic diameter was higher than that of the control group (P < 0.05). Group of white blood cell count, neutrophils/lymphocyte ratio, plasma galactose lectin-3 levels were higher than control group (P < 0.05). ROC curve analysis of left atrial appendage emptying velocity, left atrial appendage filling velocity, left atrial enddiastolic diameter and left atrial ejection fraction had higher diagnostic value (P < 0.05).

**Conclusions:**

Left atrial appendage morphology, blood flow velocity and plasma galectin-3 level are important factors to evaluate the risk of left atrial appendage thrombosis in patients with atrial fibrillation. This study improves the understanding of thrombosis, further elucidates the risk factors for thrombosis, and improves patient prognosis.

## Abstract (sr)

**Uvod:**

Cilj je bio da se istraži korelacija između morfologije dodatka levog atrijala, brzine protoka krvi i plazma galektina-3 i tromboze kod pacijenata sa atrijalnom fibrilacijom.

**Metode:**

Uključeni su pacijenti sa atrijalnom fibrilacijom koji su lečeni i obavili ultrazvučni pregled u bolnici od 2022. do decembra 2023. godine. Prema tome da li je postojala tromboza dodatka leve pretkomore, bolesnici su podeljeni u kontrolnu grupu (nije utvrđena tromboza dodatka leve pretkomore) i studijsku grupu (utvrđena je tromboza dodatka leve pretkomore). Zabeleženi su morfologija i struktura dodatka levog atrija, brzina krvotoka i nivo galektina-3 u plazmi, istražujući njegovu korelaciju sa trombozom leve pretkomore.

**Rezultati:**

Uključeno je ukupno 330 pacijenata sa atrijalnom fibrilacijom, uključujući 278 u kontrolnoj i 52 u studijskoj grupi. Leva grupa i kontrolna grupa razlika morfološke strukture (P < 0,05). Dužina glavnog režnja, područje ušća, najduži prečnik, najkraći prečnik, zapremina dodatka levog atrijala i zapremina leve pretkomode u ispitivanoj grupi bili su veći od onih u kontrolnoj grupi (P < 0,05). Brzina pražnjenja dodatka leve pretkomore, brzina punjenja i ejekciona frakcija leve komore u ispitivanoj grupi bili su niži od onih u kontrolnoj grupi, a krajnji dijastolni prečnik leve komore bio je veći od onog u kontrolnoj grupi (P < 0,05). Grupa sa brojem belih krvnih zrnaca, odnosom neutrofila/limfocita, nivoom lektina galektina-3 u plazmi je bila veća od kontrolne grupe (P < 0,05). Analiza ROC krive brzine pražnjenja dodatka leve pretkomore, brzine punjenja dodatka leve pretkomore, krajnjeg dijastolnog prečnika leve atrijalne pretkomore i ejekcione frakcije leve pretkomora imala je veću dijagnostičku vrednost (P < 0,05).

**Zaključak:**

Morfologija dodatka levog atrijala, brzina krvotoka i nivo galektina-3 u plazmi su važni faktori za procenu rizika od tromboze dodatka levog atrijala kod pacijenata sa atrijalnom fibrilacijom. Ova studija poboljšava razumevanje tromboze, dodatno razjašnjava faktore rizika za trombozu i poboljšava prognozu pacijenata.

## Introduction

Atrial fibrillation is a kind of clinical more common type of arrhythmia diseases, severe cases can cause deterioration of heart pump function. When atrial fibrillation occurs, the left atrial appendage loses its regular contraction, resulting in ineffective blood emptying. In addition, the blind end of the left atrial appendage and its internal muscle trabeculae are uneven, which is easy to produce whirlpools, and the blood flow velocity is further slowed down, leading to blood stasis and thrombosis. If in the left atrial fibrillation patients blood clots will increase the risk of cardio- cerebral appear such as thrombosis, life safety threat to patients [1] [2]. Echocardiography is a key tool in evaluating cardiac structure and function, especially on the analysis of the left - a small part of left atrium - aspects is crucial. This region is critical in patients with atrial fibrillation, a common arrhythmic symptom, because atrial fibrillation causes the heart to beat irregularly and rapidly, increasing the risk of thrombus formation in the left atrial appendage. Studying the morphology and blood flow velocity of the left atrial appendage can help to investigate how AF affects blood flow in this region. The slowing of blood flow suggests stasis in the left atrial appendage, raising the possibility of thrombosis. At the same time, the level of Galectin-3 in plasma is a biomarker related to inflammation and fibrosis, and its increase may be related to the development of cardiac diseases, especially atrial fibrillation and thrombosis. Therefore, comprehensive analysis of left atrial appendage morphology, blood flow velocity and plasma galectin-3 level is essential to understand the risk of left atrial appendage thrombosis in patients with atrial fibrillation. It can not only improve the understanding of cardiovascular risk in patients with atrial fibrillation, but also guide more personalized treatment strategies.

The left atrial appendage derives from the primordial left atrium, which is formed mainly by the adsorption of the primordial pulmonary veins and their branches. It is a finger-like projection from the main body of the left atrium. The junction is fairly well defined by a narrowing at the orifice of the appendage. There are considerable variations in its size, shape, and relationship with adjacent cardiac and extracardiac structures, which can be extremely relevant when interventional procedures are performed. In the presence of atrial fibrillation thrombus, formation often occurs within the left atrial appendage because of reduced contractility and stasis; thus, attention should be given to the left atrial appendage when evaluating and assessing patients with atrial fibrillation to determine the risk for cardioembolic complications. It is clinically important to understand left atrial appendage anatomy and function.

Left atrial thrombosis is also a contraindication for surgery. Therefore, it is necessary to accurately explore the risk factors of left atrial appendage thrombosis in atrial fibrillation [3]. The structure and function of the left atrial appendage are complex, and its contractile characteristics and endocrine function are of great significance for maintaining cardiac hemodynamic stability and homeostasis of the internal environment [4]. Previous studies have reported that many factors affect the formation of left atrial appendage thrombosis, and left atrial appendage blood flow velocity is one of the predictors. Ultrasound can display the left auricle morphological structure, thus to evaluate left auricle thrombosis in patients with atrial fibrillation [5] [6]. Plasma galectin-3 is a biological factor produced by activated macrophages, which has the effect of promoting fibrosis and plays a certain role in the occurrence and development of heart failure, myocardial fibrosis and cardiac remodeling. It may also be involved in the formation process of left atrial appendage thrombosis [7] [8]. Based on the above research background, this study aims to investigate the correlation between left atrial appendage morphology, blood flow velocity, plasma galectin-3 and left atrial appendage thrombosis in patients with atrial fibrillation.

## Materials and methods

### Study design

Patients with atrial fibrillation who received treatment and completed ultrasound examination in our hospital from 2022 to December 2023 were recorded. The patients were divided into control group (no left atrial appendage thrombosis was found) and study group (left atrial appendage thrombosis was found) according to whether there was left atrial appendage thrombosis. Inclusion criteria include: 1. by clinical comprehensive diagnosis and dynamic electrocardiogram diagnosis in patients with atrial fibrillation; 2. All patients underwent ultrasound examination and left atrial appendage thrombosis was confirmed. 3. Not undergoing cardiac surgery; 4.complete clinical data, imaging data is clear, clear results. Patients with valvular disease, cancer, or severe blood disorders may interfere with the content of the study and affect the conclusions. So exclusion criteria included: 1.atrial fibrillation due to rheumatic mitral stenosis, mitral-valve replacement, or mitral-valve repair; 2. Taking drugs that could affect the results of the study; 3. the presence of serious underlying diseases or malignant tumors; 4. severe blood system diseases or coagulopathy.

The morphology and structure of the left atrial appendage, the ultrasonic parameters of the left atrial appendage, the blood flow velocity of the left atrial appendage, and the levels of laboratory examination indicators (white blood cell count, neutrophil/lymphocyte ratio, plasma galectin-3, and hemoglobin) of the patients were recorded, and their correlation with left atrial appendage thrombosis was explored. (1) After fasting for 12 hours and undergoing esophageal supersonic examination, 2% lidocaine gel was used for pharyngeal infiltration anesthesia. The examination began approximately 10 minutes after the onset of anesthesia. (2) The examination was conducted using the Philips EPIQ7C ultrasonic diagnostic instrument with an X7-2 t probe operating at frequencies ranging from 2 to 7 MHz. The examination involved observing various (0°, 45°, 90°, 135°) of the structure, measuring the length, open area, inner diameter, and depth of the left main leaf. (3) Real-time three-dimensional images of the left atrium were obtained using QLab software and 3Dq plug-ins, capturing parameters such as the longest diameter, shortest diameter, volume, and morphological structure (e.g., chicken wings, vane, cauliflower, cactus form). (4) The left atrial appendage and mitral valve were visualized in the two-chamber cardiac view within the esophagus, ensuring that the entire left atrium and left atrial appendage were centered in the field of view. (5) The area of the left atrial appendage was measured in each cross-sectional section, and the left atrial appendage ejection fraction (LAAEF) was calculated using the formula: (maximum area of the left atrial appendage – minimum area of the left atrial appendage)/maximum area of the left atrial appendage × 100%. (6) The left atrial appendage end-systolic volume and end-diastolic volume were computed based on the obtained sections. Pulse Doppler detection was utilized to examine the left auricle blood flow spectrum, assessing parameters such as the emptying rate and filling speed across three consecutive cardiac cycles, with the average value being recorded as the final result. Fasting venous blood of 3 mL was drawn from the subjects in the morning, and the plasma was separated after anticoagulation. Using Beckman AU5800 automatic biochemical analyzer determination of TC, TG, HDL and LDL levels. The hemoglobin content was detected by automatic blood cell analyzer and colorimetric method. Using enzyme-linked immunosorbent assay method of determination of plasma galactose lectin-3 levels.

This study was approved by the ethics committee. Signed written informed consents were obtained from the patients and/or guardians.

### Statistical analysis

Statistic Package for Social Science (SPSS) 23.0 software (IBM, Armonk, NY, USA) was used for data processing. The chi-square test is applicable to the analysis of categorical information. Rank sum test (rank sum test), which is a non-parametric test. It does not depend on the specific form of the overall distribution and can be applied without regard to what kind of distribution the subject is and whether the distribution is known, thus making it more practical. t-test is applicable to normally distributed data. The ROC curve is a graphical tool used to demonstrate the performance of a diagnostic system or any process that needs to differentiate between the categories of a normal distribution. It shows the accuracy of diagnostic results by plotting line plots of sensitivity and specificity and provides a visual way to evaluate and compare different diagnostic methods or models. In this paper, appropriate statistical methods were selected based on the characteristics of the data. The count data such as patient’s gender and past medical history were analyzed by chi-square test, and the morphology and structure of left atrial appendage were analyzed by rank sum test, which were expressed as (n/%). Measurement data such as left the ultrasonic parameters, such as blood flow velocity and laboratory indexes in (plus or minus s) said, independent sample t-test between groups; ROC curve was used to explore the diagnostic value of the above indicators for the presence of left atrial appendage thrombosis.<br>=0.05 was considered as the test level.

## Results

**Figure 1:**
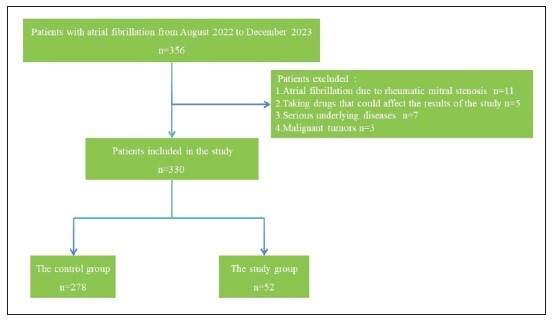
Hologram technology can specifically observe renal blood vessels, renal pelvis and renal lamps.

From August 2022 to December 2023, 356 patients with atrial fibrillation were treated in our hospital and completed the ultrasound examination, and 330 patients were finally included in the study. Among them, 278 patients in the control group were not found to have left atrial appendage thrombosis, and 52 patients in the study group were found to have left atrial appendage thrombosis. Study flow diagram can be seen in Figure 1. General data comparison between groups had no difference (P > 0.05). Details can be found in Table 1.

**Table 1:** Comparison of general data between the two groups (x̄±s, n/ (%)).

Group	Age (years)	Male/Female	BMI (kg/m^2^)	Persistent Atrial<br>Fibrillation
Control Group (n=278)	56.26±12.55	201/77	22.45±3.45	87 (31.29)
Study Group (n=52)	58.14±13.56	44/8	22.64±3.15	16 (30.77)
χ2/t	-0.979	3.473	-0.369	0.006
P	0.328	0.062	0.712	0.940
Group	Coronary Heart Disease	Hypertension	Diabetes	Duration (months)
Control Group (n=278)	33 (11.87)	164 (58.99)	35 (12.59)	12.02±5.46
Study Group (n=52)	6 (11.54)	26 (50.00)	5 (9.62)	11.87±4.98
χ2/t	0.005	1.450	0.364	0.184
P	0.946	0.228	0.546	0.854

**Figure 2:**
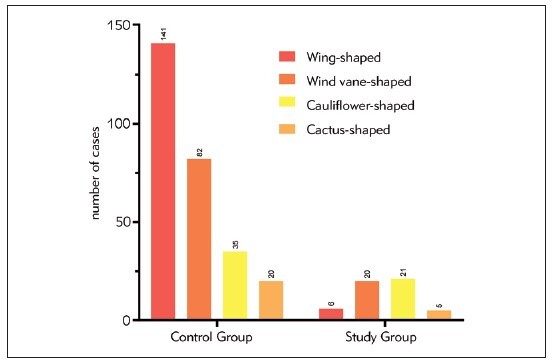
Left Atrial Appendage Morphology.

After examination, there were differences in the morphological structure of the left atrial appendage between the study group and the control group: the proportion of wind vanness, cactus and cauliflower shapes in the study group was higher than that in the control group, and the proportion of chicken wing shapes was lower than that in the control group (P < 0.05). Details can be found in Table 2, Figure 2.

**Table 2:** Comparison of Left Atrial Appendage Morphology (n/%).

Group	Wing-shaped	Wind vane-shaped	Cauliflower-shaped	Cactus-shaped
Control Group (n=278)	141 (50.72)	82 (29.50)	35 (12.59)	20 (7.19)
Study Group (n=52)	6 (11.54)	20 (38.46)	21 (40.38)	5 (9.62)
Z	5.445			
P	0.000			

The echocardiographic parameters of the left atrial appendage were compared: the main lobe length, ostial area, longest diameter, shortest diameter, left atrial appendage volume and left atrial volume in the study group were higher than those in the control group (P < 0.05). Details can be found in Table 3.

**Table 3:** Comparison of Various Ultrasound Parameters of the Left Atrial Appendage (x̄±s).

Group	Left Atrial Appendage Main<br>Leaf Length (mm)	Left Atrial Appendage<br>Orifice Area (mm^2^)	Left Atrial Appendage<br>Longest Diameter (mm)
Control Group (n=278)	41.44±3.56	433.65±52.28	29.55±3.45
Study Group (n=52)	44.58±3.02	477.26±55.15	31.02±3.16
t	-5.969	-5.473	-2.856
P	0.000	0.000	0.005
Group	Left Atrial Appendage<br>Shortest Diameter (mm)	Left Atrial Appendage<br>Volume (mm^3^)	Left Atrial<br>Volume (mm^3^)
Control Group (n=278)	19.22±3.46	6.55±1.02	76.55±11.45
Study Group (n=52)	20.66±3.08	7.11±1.08	80.22±13.87
t	-2.800	-3.600	-2.048
P	0.005	0.000	0.041

In the comparison of left atrial appendage blood flow velocity, the left atrial appendage emptying velocity, filling velocity and left atrial ejection fraction in the study group were lower than those in the control group, and the left atrial end-diastolic diameter was higher than that in the control group (P < 0.05). Subsequently, we counted the differences in atrial appendage blood flow velocities among the three concomitant diseases, coronary artery disease, hypertension, and diabetes, and found that the left atrial appendage emptying velocity, filling velocity, and left atrial ejection fraction were lower in the study group than in the control group and that left atrial end-diastolic diameter was higher in the study group than in the control group. Details in Table 4.

**Table 4:** Comparison of Left Atrial Appendage Blood Flow Velocities (x̄±s).

Group		Left Atrial<br>Appendage	Left Atrial<br>Appendage Filling	Left Atrial End-<br>Diastolic Diameter	Left Atrial Ejection Fraction<br>(%)	
Control Group (n=278)		49.89±7.01	44.49±6.88	46.18±2.16	61.23±2.56	
Study Group (n=52)		35.45±8.45	34.55±7.49	48.56±2.08	58.23±1.99	
t		13.178	9.428	-7.334	8.006	
P		0.000	0.000	0.000	0.000	
Group		Left Atrial<br>Appendage<br>Emptying Velocity<br>(cm/s)	Left Atrial<br>Appendage Filling<br>Velocity (cm/s)	Left Atrial End-<br>Diastolic Diameter<br>(mm)	Left Atrial Ejection<br>Fraction (%)	P
Coronary<br>Heart Disease	Control Group	49.63±6.25	45.39±7.20	47.16±1.98	60.23±2.34	0.000
	Study Group	34.22±5.29	33.42±8.21	52.23±2.12	56.13±1.55	
Hypertension	Control Group	48.27±7.26	46.36±6.23	46.18±2.02	61.28±2.21	0.000
	Study Group	33.12±8.02	34.19±2.05	53.26±1.02	54.23±1.23	
Diabetes	Control Group	50.12±1.56	44.25±7.96	48.55±2.29	61.28±2.57	0.000
	Study Group	33.58±6.05	32.50±1.46	54.25±1.03	55.46±1.69	

In laboratory tests, There were no significant differences in the levels of hemoglobin, triglyceride, total cholesterol, high-density lipoprotein cholesterol and low-density lipoprotein cholesterol between the two groups (P>0.05). The white blood cell count, neutrophil/lymphocyte ratio and plasma galectin-3 level in the study group were higher than those in the control group (P < 0.05). Details in Table 5.

**Table 5:** Laboratory Index Detection (x̄±s).

Group	White Blood Cell<br>Count (×10^9^/L)	Neutrophil/Lymphocyte<br>Ratio	Plasma Galactose-3<br>(ng/mL)	Hemoglobin (g/L)
Control Group	6.11±1.02	1.98±0.49	13.22±2.65	140.23±15.46
Study Group (n=52)	6.45±1.35	2.33±0.56	16.78±5.11	142.56±13.68
t	-2.088	-4.619	-7.455	-1.015
P	0.038	0.000	0.000	0.311
Group	Triglycerides (mmol/L)	Total Cholesterol<br>(mmol/L)	LDL Cholesterol<br>(mmol/L)	HDL Cholesterol<br>(mmol/L)
Control Group	1.26±0.35	4.12±0.56	2.65±0.71	1.23±0.24
Study Group (n=52)	1.24±0.32	4.15±0.77	2.66±0.81	1.18±0.27
t	0.383	-0.332	-0.091	1.351
P	0.702	0.740	0.927	0.178

**Figure 3:**
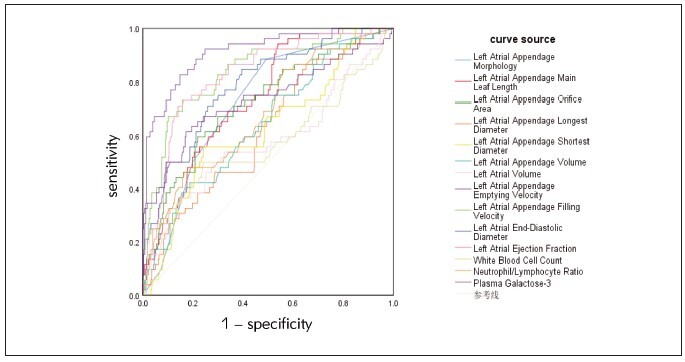


To evaluate the diagnostic value of left atrial appendage related parameters and laboratory parameters in patients with left atrial appendage thrombosis ROC curve analysis was showed that: Left heart left ear shape, the leaf length, left open area, the longest diameter, left the shortest diameter, left auricle volume, left auricle emptying rate, filling rate, left ventricular end-diastolic diameter, left ventricular end-diastolic diameter, neutrophils/lymphocyte ratio, plasma galactose lectin-3 patients with diagnosis of left atrium thrombus AUC value points Don’t: 0.723, 0.740, 0.720, 0.618, 0.624, 0.641, 0.913, 0.840, 0.785, 0.827, 0.678, 0.735, The diagnostic value of left atrial appendage emptying velocity, left atrial appendage filling velocity, left atrial end-diastolic diameter and left atrial ejection fraction was higher (P < 0.05). Details can be found in Table 6, Figure 3.

**Table 6:** Diagnostic value of left atrial appendage related parameters and laboratory parameters for patients with left atrial appendage thrombosis

Index	AUC	Optimal<br>cutoff value	95%CI		Sensitivity	Specificity	P
			Lower limit	Upper limit			
Left Atrial Appendage Morphology	0.723	Wind vane-shaped	0.655	0.791	0.885	0.507	0.000
Left Atrial Appendage Main Leaf<br>Length	0.740	40.825	0.676	0.805	0.942	0.480	0.000
Left Atrial Appendage Orifice Area	0.720	472.77	0.642	0.798	0.596	0.781	0.000
Left Atrial Appendage Longest<br>Diameter	0.618	28.135	0.538	0.699	0.865	0.449	0.007
Left Atrial Appendage Shortest<br>Diameter	0.624	21.655	0.539	0.709	0.558	0.769	0.005
Left Atrial Appendage Volume	0.641	7.370	0.560	0.722	0.423	0.813	0.001
Left Atrial Volume	0.578	89.995	0.482	0.674	0.327	0.869	0.075
Left Atrial Appendage Emptying<br>Velocity	0.913	42.970	0.870	0.957	0.827	0.853	0.000
Left Atrial Appendage Filling<br>Velocity	0.840	37.735	0.779	0.901	0.731	0.842	0.000
Left Atrial End-Diastolic Diameter	0.785	47.620	0.718	0.851	0.712	0.741	0.000
Left Atrial Ejection Fraction	0.827	58.515	0.767	0.886	0.712	0.860	0.000
White Blood Cell Count	0.576	6.810	0.476	0.675	0.500	0.769	0.084
Neutrophil/Lymphocyte Ratio	0.678	2.455	0.598	0.759	0.865	0.496	0.000
Plasma Galactose-3	0.735	15.800	0.647	0.823	0.615	0.827	0.000

## Discussion

When af, the inner diameter of the left as the left atrial pressure increases, the left auricle structure change. The structural changes of the left atrial appendage are reflected in the loss of effective contractility, irregular fibrillation, decreased pumping function, and impaired blood emptying, which eventually lead to blood stasis and thrombosis [9] [10]. Evaluation of atrial fibrillation patients with thrombosis is an important link to guide clinical treatment and improve the prognosis of patients. This study was performed in patients with atrial fibrillation and biochemical indicator detection, left to explore the ultrasonic morphology structure and blood flow velocity and plasma galactose lectin-3 and the correlation of thrombosis in left atrial fibrillation. Previous studies have examined only blood flow velocity, but have not discussed the effects of galectin-3 and LAA morphology on thrombosis. This study not only discusses the effect of the morphology of the LAA on thrombosis, but also incorporates blood flow velocity indices, white blood cell counts, and plasma galactoglucan lectin-3 levels to predict outcomes. The importance of LAA morphology and galectin-3 on thrombosis was emphasized.

The results of this study showed that there were differences in the morphological structure of the left atrial appendage between the study group and the control group. The proportion of wind vanes, cactus, and cauliflower shapes in the study group was higher than that in the control group, and the proportion of chicken wing shapes was lower than that in the control group (P < 0.05). This phenomenon is related to the anatomical structure of the left atrial appendage. Vane shape, cacti and cauliflower shape left comb muscle is more abundant. Some studies have found that the cauliflower shape has the strongest association with thrombotic events, and the cauliflower shape has a greater depth of the left atrial appendage and a greater content of the pectineus muscle, which is conducive to blood block. Internal organization and cauliflower shape structure course is disorder, shorter diameter shaft, and larger quantity of lobules thrombosis risk big [11]. Compared with other types of LAA, cauliflower LAA has more branches and more complex internal structure, which is more likely to lead to vortex in the LAA and thrombosis. The number of “cactus like” main leaflets is large, and the secondary leaflets are formed from the upper and lower parts. The variation of leaflets at each level is strong, and the tortuosity of the left atrial appendage is easy to increase, which makes the blood block here and provides conditions for the formation of thrombosis [12]. “Barometer” have a backbone lobule, but its position variation, and secondary lobule and triple the number of lobular have larger changes, easy to make left bending, blood easy sedimentation in the formation of blood clots [13]. The “chicken wing-like” main leaflet can fold itself, which has a shorter LAA depth and the lowest risk of thrombosis. Transesophageal echocardiography can be used to evaluate the structure of left atrial appendage, which can provide more information for clinical diagnosis of left atrial appendage thrombosis. All of these studies are consistent with the conclusion of this study. Previous studies also have found that Velocities >40 cm/s are suggestive of adequate flow within the appendage and a low risk for thrombus formation. Color Doppler to a low Nyquist limit can aid in visualization of flow within the LAA. E/e’ and e’ velocity were found to be independently associated with an LAA thrombus in patients with nonvalvular AF.

The results of this study showed that the main lobe length, orifice area, longest diameter, shortest diameter, left atrial appendage volume and left atrial volume in the study group were significantly higher than those in the control group (P < 0.05). In patients with atrial fibrillation and left atrial appendage thrombosis, the left atrial appendage is easily pulled by the left atrial, the area and volume of the left atrial appendage are increased, the left atrial ejection function is weakened, the emptying ability is limited, and the blood is prone to stasis and thrombosis [14] [15]. And left the leaf length, open area, the longest diameter, the shortest path left longer makes structure is long and narrow, bending, easily occur in this case the blood sedimentation, provides conditions for thrombosis.

This study shows that: the team left the emptying speed, filling rate, left ventricular ejection fraction were lower than the control group, and higher left entricular end-diastolic diameter than the latter. It has been confirmed by previous studies [16] that both the filling velocity and the emptying velocity of the left atrial appendage have a certain correlation with the formation of left atrial appendage thrombosis, and this study also obtained consistent results, and this study also found that the above indicators are risk factors for the formation of left atrial appendage thrombosis. Differences between control and study groups for three concomitant diseases, coronary artery disease, hypertension, and diabetes mellitus, indicate that all three diseases are high risk factors for thrombosis. Left with independent, and diastolic function of left auricle can buffer left atrial contraction capacity and pressure changes caused by a series of changes. The filling state of the LV also plays an important role in maintaining left atrial appendage function. Cardiac diastolic dysfunction in patients with atrial fibrillation, left atrial emptying ability to drop, left atrial volume index increases, in turn, increased pressure inside the room, left auricle blood in turbulent state, left a vicarious expansion, intimal fibrosis, myocyte hypertrophy, and appear the phenomenon such as apoptosis [17]. The above phenomenon makes the chance of platelet and endothelial contact, makes the platelet activation in great quantities, increase the risk of blood clots in the left atrium. In the left auricle with comb structure, when it is left atrial stretch, including the blood flow velocity slowed, also prone to blood clots.

According to the results of this study team left atrial ejection fraction is lower than the control group, left atrial end-diastolic diameter is higher than the latter. Prompt ultrasound can be showed that left ventricular dysfunction in patients with atrial fibrillation and left prediction risk of blood clots. Left atrial ejection fraction (LVEF) and left atrial end-diastolic diameter (LVEDD) are commonly used to evaluate the function of left atrial appendage. When the function of left atrial appendage is weakened and the left atrial appendage function is weakened, the above indexes change, the left atrial blood flow is stagnant, and blood is retained here, leading to thrombosis. Hematological indicators including white blood cell count, neutrophil/lymphocyte ratio, and plasma galectin-3 level were also analyzed in this study, and the results showed that the levels of these indicators were higher in the study group. Inflammatory cytokine played an important role in the process of thrombosis. It has been reported [18] that white blood cell and neutrophil/lymphocyte ratio are significantly positively correlated with left atrial volume index and affect the process of atrial remodeling. Combined with the results of this study, in patients with AF, inflammation leads to endothelial injury and dysfunction, promotes the hypercoagulable state of AF, and promotes the formation of left atrial appendage thrombosis.

This study also found that galactose lectin-3 is a risk factor for left atrium thrombus in patients with atrial fibrillation. Traditional risk factors for atrial fibrillation (such as hypertension, coronary artery disease and diabetes) can stimulate macrophages release galactose lectin-3, then promote myocardial tissue inflammation cell aggregation and activation, leading to cardiac fibrosis. And myocardial fibrosis can stimulate macrophage activation, form a vicious circle, and make atrial fibrillation patients plasma galactose lectin - three levels rising further [19]. Combined with the analysis of the results of this study, the increased level of galectin-3 promotes the process of myocardial fibrosis, affects atrial function, and indirectly promotes the formation of left atrial appendage thrombosis. It should be noted that the occurrence of thromboembolism is influenced by many factors such as age, hypertension, and diabetes. Galactose lectin-3 levels in evaluating left thromboembolic events in terms of value validation need further research. The formation of left atrial appendage thrombosis in patients with atrial fibrillation is a process involving multiple factors, which may be related to atrial remodeling, inflammation, platelet activation, increased thrombin, and other factors such as changes in left atrial appendage structure, function, and hemodynamic changes [20]. In the future, we further validated our conclusions by carefully delineating the morphological structure of LAA and predicting the risk of thrombosis in patients using machine learning in conjunction with galectin-3 levels.

There are still some shortcomings in this study. The study group was relatively small compared to the control group, and we will increase the size of the study group in future studies. The irregular anatomical structure of the left atrial appendage may lead to errors in measuring the data of the left atrial appendage. There are subjective factors in the judgment of the change of ultrasonic beam reflection velocity, which needs further research and improvement. Left the place on put together is narrated, ultrasonic morphology and blood flow velocity and plasma galactose lectin - is 3 level assessment of left atrial fibrillation risk of blood clots, the important factors that can be used for clinical diagnosis and treatment decisions.

## Dodatak

### Conflict of interest statement

All the authors declare that they have no conflict of interest in this work.
